# Ozone Decontamination of Medical and Nonmedical Devices: An Assessment of Design and Implementation Considerations

**DOI:** 10.1021/acs.iecr.2c03754

**Published:** 2023-03-01

**Authors:** Emmanuel I. Epelle, Andrew Macfarlane, Michael Cusack, Anthony Burns, Jude A. Okolie, Parag Vichare, Luc Rolland, Mohammed Yaseen

**Affiliations:** † School of Computing, Engineering & Physical Sciences, University of the West of Scotland, Paisley PA1 2BE, United Kingdom; ‡ ACS Clothing, 6 Dovecote Road Central Point Logistics Park, Centralpark ML1 4GP, United Kingdom; § Gallogly College of Engineering, University of Oklahoma, Norman, Oklahoma 73019, United States of America

## Abstract

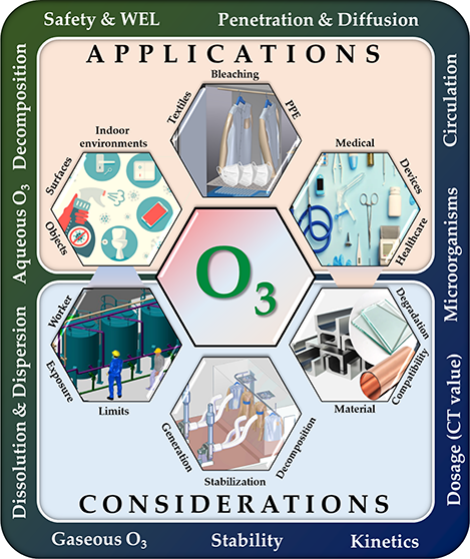

The control of infectious diseases can be improved via carefully designed decontamination equipment and systems. Research interest in ozone (a powerful antimicrobial agent) has significantly increased over the past decade. The COVID-19 pandemic has also instigated the development of new ozone-based technologies for the decontamination of personal protective equipment, surfaces, materials, and indoor environments. As this interest continues to grow, it is necessary to consider key factors affecting the applicability of lab-based findings to large-scale systems utilizing ozone. In this review, we present recent developments on the critical factors affecting the successful deployments of industrial ozone technologies. Some of these include the medium of application (air or water), material compatibility, efficient circulation and extraction, measurement and control, automation, scalability, and process economics. We also provide a comparative assessment of ozone relative to other decontamination methods/sterilization technologies and further substantiate the necessity for increased developments in gaseous and aqueous ozonation. Modeling methodologies, which can be applied for the design and implementation of ozone contacting systems, are also presented in this review. Key knowledge gaps and open research problems/opportunities are extensively covered including our recommendations for the development of novel solutions with industrial importance.

## 1 Introduction

The critical role played by contaminated fomites in the spread of diseases over a myriad of environments (hospitals, offices, laboratories, and schools) necessitates the application of surface disinfection methods for the prevention of infections.^1,2^ The wide variation in the scale of the disinfection procedure (from small surfaces to huge environments) has also led to several engineering developments for the efficient application of numerous chemical disinfectants.^3^ One of such disinfectants is ozone (a potent antimicrobial agent), which has received tremendous research interest for decontamination, since the advent of the COVID-19 pandemic.^4−6^ Its excellent oxidation potential (2.07 V) and rapid decomposition into oxygen make it particularly attractive and versatile for not only decontamination but also bleaching and deodorization in both air and water.^7,8^ This multifunctional attribute also makes it widely applicable in different industries including pulp and paper, textile processing, aquaculture, drinking water treatment, animal husbandry, wastewater treatment, food, healthcare (therapeutic applications), and medical equipment processing.^9−18^ However, its highly unstable property implies that it cannot be stored for subsequent applications and must be generated on-site for immediate use. A key area requiring the application of ozone is that of medical device sterilization.

Currently, ethylene oxide is the most commonly used sterilization method in the US medical sector, accounting for ∼50% of all devices requiring sterilization.^19^ However, ethylene oxide is carcinogenic and there are concerns regarding the release of unsafe levels of ethylene oxide into the environment. These issues have led to several initiatives and innovation challenges by the US Food and Drug Administration (FDA), which are geared toward the development of novel sterilization methods, for the replacement of ethylene oxide.^20^ Ozone has considerable potential to replace this disinfectant. Although the antimicrobial properties of ozone have been known for decades, it has mainly been utilized for the removal or degradation of pollutants in waste and drinking water. Its application for the decontamination of medical and nonmedical devices is relatively new and has not been adequately explored. Furthermore, the cost of ozone production has dropped significantly over the last 2 decades, and this has paved the way for new lab-scale and industrial developments.^21^ Nonetheless, a majority of these developments have been at a small scale, and there are several knowledge gaps surrounding the implementation of ozone decontamination systems at a large scale.

To the best of the authors’ knowledge, many reviews on the application of ozone are application specific, focusing on water/wastewater treatment,^22,23^ aquaculture,^24^ food decontamination,^11,25−28^ textile processing,^29^ medical device sterilization,^30,31^ pulp and paper processing,^10^ biomass processing,^32^ ozone therapy,^33^ and SARS-CoV-2 (COVID-19) inactivation.^8,34^ In this review, we present and discuss the current state of the art on the implementation of ozone decontamination systems, and draw applicable inferences from lab-scale studies that aid the industrial design and installation of ozonation systems. First, the uniqueness of ozone, and why it is becoming the preferred decontamination method (for surface sterilization), in comparison to others is presented; thereafter, engineering considerations regarding material compatibility, ozone generation and decomposition, circulation and extraction, measurement and control, scalability and flexibility of operation (in gaseous and aqueous forms), automation, health and safety, and the economics of ozonation processes are discussed. We also highlight key mathematical models that can aid the sizing of ozone contact equipment, and provide our recommendations on the way forward/future directions from both academic research and industrial application perspectives. In comparison to other reviews in the field of ozonation, this is the first review to simultaneously consider these key aspects of ozone systems design and implementation. It is worth mentioning that in this review we pay particular attention to gaseous ozone application while providing some insights into its aqueous application. It is hoped that this review will appeal to a broad range of scientific communities for the continued development of ozone-related technologies.

## 2 How Ozone Compares to Other Sterilization Methods

Besides ozone, several studies have demonstrated the effectiveness of a variety of sterilization methods including cold plasma, gamma irradiation, ultraviolet irradiation (of type C), dry and moist heat, steam, hydrogen peroxide (gas and liquid) microwave, peracetic acid, ethanol, glutaraldehyde, orthophthalaldehyde (OPA), ethylene oxide, benzalkonium chloride, and hypochlorite. The performance of these methods for diverse applications has mainly been assessed using factors such as decontamination efficacy, cycle time, penetration capability, substrate/material compatibility, operational safety, cost of implementation, and environmental sustainability, with an overwhelming majority focusing on the decontamination efficacy. It should be highlighted that the cycle time in this review represents the sum of the generation time (or time required to attain the desired sterilant concentration in a chamber/vessel), the contact time at the desired concentration, and the decomposition time to concentrations below the safety limit.

**Figure 1:**
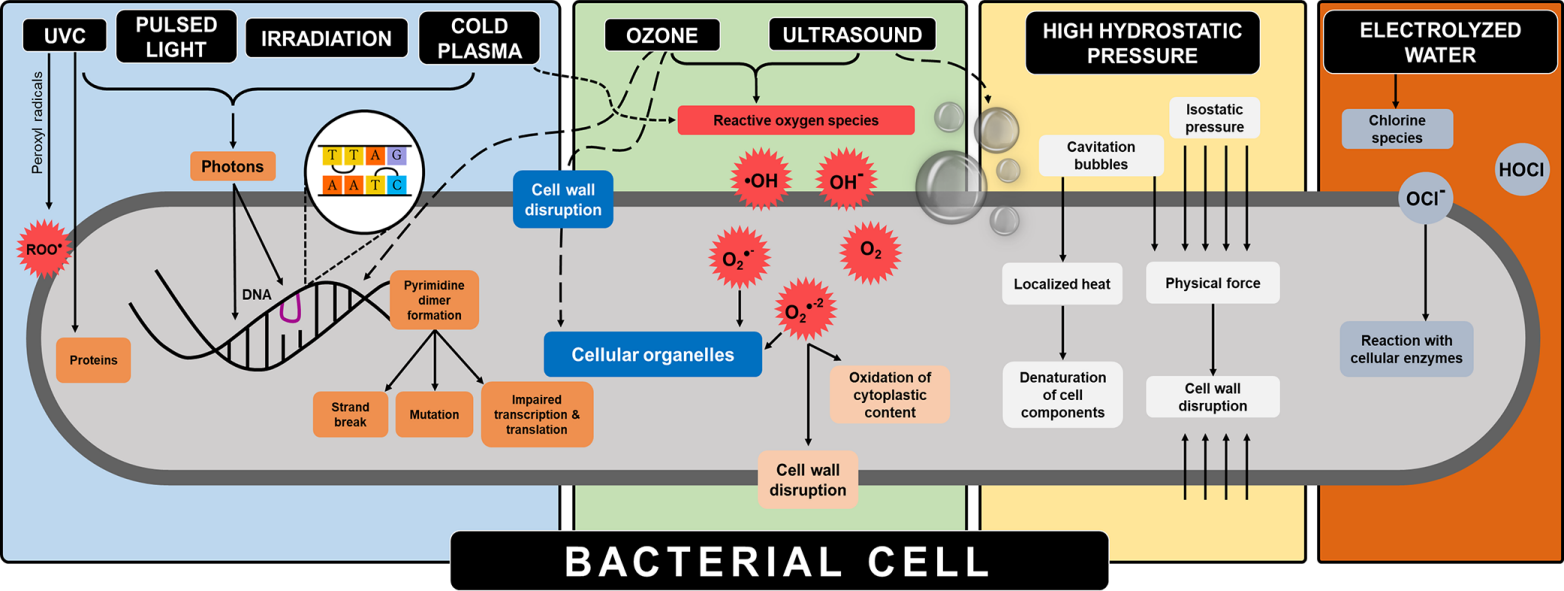
Comparative representation of the inactivation mechanisms of selected disinfection methods (adapted from refs (38 and 39)).

Table 1 presents a summary of some of the reported merits and demerits of these sterilization methods. Methods that rely on full immersion of the object/device/material to be disinfected (e.g., glutaraldehyde) tend to be less preferred due to the postdisinfection steps that are required before they can be reused. Thus, the applicability of a disinfectant in the gaseous form or via misting (e.g., ozone, hydrogen peroxide or peracetic acid^35−37^) is a favorable attribute that reduces the need for long drying procedures after the main disinfection phase. In addition to ozone, supercritical CO_2_ and nonthermal plasma are emerging methods that require further development. Other methods that are sparingly utilized in isolation, but not captured in Table 1, include high hydrostatic pressure, pulsed light, ultrasound, and electrolyzed water;^38^ however, these have immense antimicrobial properties, particularly during hybrid application with other well-known/established methods. Figure 1 demonstrates key differences in the inactivation mechanisms of selected disinfection methods during contact with a bacterial cell.

**Table 1:** Some Advantages and Disadvantages of Popularly Applied Disinfection Methods^a^

disinfection method	advantages	disadvantages	ref
Ozone	• Flexible application (low concentrations for longer durations, usually give the same effect as high concentrations for shorter times).	• Relatively lower compatibility with some polymers, whereas the compatibility of some is still unknown (Table 2).	(4, 8, 38, 43, 46, 47)
	• Environmentally friendly (decomposes to O_2_).	• May produce bromates as a disinfection byproduct, in the presence of bromine (at high concentrations) during aqueous applications.	
	• Excellent for disinfecting heat-sensitive materials.	• Large-scale generation costs can be high since it is difficult to store; may also pose a fire risk, particularly in oxygen-rich environments.	
	• Can be readily applied as a gas, in aqueous form, and via suspended mists.		
	• Excellent penetration into hard-to-reach areas of an object.		
	• Effective against a wide range of organisms (sporicidal and virucidal); no activation required.	• Inhalation causes shortness of breath, asthma, heaviness of the chest, dry throat, cough and headaches; long-term chronic exposure may be fatal.	
			
UVC	• Particularly effective against different fungi.	• Poor penetration, particularly when materials of complex geometries are to be disinfected.	(5, 48)
	• Applicable in dry (air) and wet (water) conditions.		
	• Effective against a wide range of organisms; sporicidal and virucidal.	• Effectiveness decreases as the distance from the UVC source increases.	
		• High installation costs.	
		• Induces color change, and embrittlement of some plastics.	
		• Exposure causes severe eye and skin irritation.	
			
Chlorine-based methods	• Good microbiocidal activity	• Nonsporicidal.	(6, 49, 50)
	• Inexpensive and commonly used in surface cleaning and bleaching agents.	• Harmful disinfection byproducts are formed.	
		• Slower inactivation kinetics compared to ozone.	
		• Degradation of stainless steel and certain plastics.	
			
H_2_O_2_	• Better mater material compatibility compared to ozone.	• Incompatible with some materials as shown in Table 2; metals such as silver, copper, brass, and zinc may be affected.	(36, 46, 51−53)
	• Effective against a broad range of microorganisms; also sporicidal.		
	• Readily applicable in gaseous and aqueous forms.	• An irritant to the eyes upon exposure.	
	• Excellent for heat-sensitive materials	• Inhalation causes shortness of breath; long-term chronic exposure may be fatal.	
	• No activation is required.		
	• Nonstaining	• Its instability implies an increased cost for large-scale continuous generation.	
			
Ethylene oxide	• Excellent compatibility with a wide range of polymers (see Table 2).	• Carcinogenic.	(48, 54)
	• Good penetration capabilities (particularly medical packaging).	• Toxic, flammable, and explosive.	
	• Good antibacterial properties.	• Longer cycle times are required.	
		• High cost of operation.	
		• Higher pressures than atmospheric pressure are typically required.	
		• Requires extensive aeration to remove excess EtO.	
			
Steam	• Nontoxic.	• Unsuitable for heat-sensitive materials.	(55−57)
	• Good penetration into medical devices.	• Need for drying after treatment due to wetness of procedure.	
	• Easy to implement and control.	• May induce rust in equipment.	
			
Surfactants, quaternary ammonium compounds	• Possesses good detergent properties.	• Not sporicidal.	(58)
	• Does not cause irritation.	• May cause occupational asthma.	
	Good surface disinfectant for noncritical items.	• May require full immersion for effective penetration.	
			
γ Radiation	• Excellent penetration.	• May induce polymer weakening and further degradation.	(57)
	• Effective for sterilizing single-use medical materials.	• Not effective for reusable materials.	
	• Rapid sterilizability.	• High capital cost requirements.	
			
Alcohols	• Nonstaining.	• Nonsporicidal.	(5, 48, 58)
	• Wide-ranged antibacterial activity.	• Ineffective against some viruses.	
	• Inexpensive.	• Can be flammable.	
			
Peracetic acid	• Effective against a wide range of organisms (also sporicidal).	• Can cause severe irritation to the eyes and skin.	(58−60)
	• Applicable for fogging purposes.	• Corrosive to metals.	
	• Activation not required.	• High operational costs.	
	• Good material compatibility.	• Medical instruments may require total immersion.	
		• Difficult sterile storage.	
			
Glutaraldehyde	• Relatively lower cost.	• Activation required.	(58, 60)
	• Good material compatibility.	• Strong odor, which causes respiratory irritation.	
		• Slow bactericidal activity.	
			
Ortho-phthalaldehyde	• No activation required.	• Expensive.	(59−61)
	• Rapid disinfecting action.	• Slow sporicidal activity.	
	• Insignificant odor compared to glutaraldehyde.	• Stains materials and surfaces.	
			
Supercritical CO_2_	• Good penetration capability.	• Expensive equipment for implementation.	(62−64)
	• Effective for heat-sensitive materials.	• Very sensitive to pressure and temperature conditions.	
	• Excellent antimicrobial properties.	• May require other chemical additives.	
		• May compromise the functional properties of materials (e.g., the filtering and breathability properties of facemasks).	
			
Cold atmospheric or nonthermal plasma	• Excellent penetration since it works at the atomic/molecular level.	• High installation/investment costs.	(65−74)
	• Extensive application for a wide range of microorganisms (excellent fungicidal properties).	• Typical small volumes of generated plasma limit its application on a large scale (involving many samples or samples with a large surface area).	
	• Fast-acting disinfection method.	• Limited to batch-based application.	
	• Excellent therapeutic properties (for treatment of chronic wounds).	• There are concerns regarding its penetration efficiency.	
	• Environmentally friendly.	• Its adaptability to different scenarios is still an open research question.	
		• Enumeration of the dosage may be difficult.	
			
Metallic nanoparticles	• Can be used to functionalize or coat materials and surfaces, embedding them with antimicrobial properties.	• Intensive preparatory procedures.	(75−78)
	• Nontoxic.	• Limited antimicrobial properties.	
	• Effective at low concentrations.	• May require chemical additives to give desired antimicrobial properties.	

a A comparison of the exposure limits of the chemical disinfectants is presented in Table 5.

The direct attack on the DNA, cell wall disruption, and subsequent oxidation, diffusion and reaction with key cell constituents, and the creation of localized heat are key attributes of these methods. Ethylene oxide (EtO) is one of the most commonly applied methods for the terminal sterilization of medical devices, and this is due to the efficient microbial inactivation, excellent compatibility with a broad range of materials, and the reasonable cost of implementation. Heat-based sterilization methods are usually not compatible with several polymeric components of medical devices that cannot withstand high temperatures. Unlike ozone, which has to be generated on demand, EtO is usually pressurized and stored in liquid form for use in sterilization plants. Its inactivation mechanism is the alkylation of the amine groups of microbial DNA.^40^ For efficient EtO application, a validated combination of humidity, gas concentration, temperature, and exposure duration must be utilized.^41^ Vacuum cycles are typically employed to increase the penetration of the gas into the substrate to be decontaminated. After treatment (which could last from 6 h to several days),^42^ EtO concentrations can be brought below the permissible limits via vacuum purging and aeration. While there are several similarities between EtO’s application and that of gaseous ozone (particularly the necessity of a humid environment for efficient inactivation), EtO poses a severe health and safety risk (a human carcinogen), as documented in Table 1. Its replacement with more environmentally friendly methods such as ozone and hydrogen peroxide has been a key subject of growing recent interest in the last 5 years. However, it should be noted that long-term chronic exposure to ozone can cause lung damage, and asthma and could be fatal.^43^ Similarly, the inhalation of hydrogen peroxide causes irritation to the lungs and shortness of breath. Higher and long-term exposures may lead to the buildup of fluids in the lungs (pulmonary edema), bronchitis, and even mortality (resulting from oxygen embolism).^44^ Thus, adequate control systems are required in facilities where these alternative decontamination techniques are applied to prevent uncontrolled exposure and to ensure safe working conditions. Further details of the exposure limits of different gaseous disinfectants are presented in Section 3.7.

Furthermore, the compatibility of key disinfection methods with commonly applied polymers as shown in Table 2 is a key differentiator affecting the applicability in different industries. Table 2 also illustrates why ethylene oxide has been predominantly applied for the sterilization of medical equipment, despite its toxicity. Relative to other methods, it has the best compatibility with a wide range of polymers. The high reactivity of ozone, coupled with its powerful oxidizing capabilities, makes it a suitable candidate for the rapid sterilization of medical devices. However, a typical drawback of EtO is the long cycle time required for sterilization. Besides these benefits, ozone’s (gas) ability to decontaminate items without leaving residues, coupled with its spontaneous decomposition to oxygen, makes it more environmentally friendly compared to ethylene oxide. Furthermore, since ozone gas must be generated on demand, storage and transportation requirements are minimal, thus reducing operational costs compared to EtO. Although not directly applicable to gaseous sterilization of medical devices with ozone, it is important to highlight that the application of aqueous ozone may induce bromate formation (a toxic disinfection byproduct) in waters with a high bromine content during water treatment. Furthermore, the degradation of ozone-incompatible materials may produce other harmful compounds. Thus, it is necessary to ensure that the devices to be sterilized are composed of ozone-compatible materials. While a direct comparison of the microbial inactivation efficiencies of EtO and ozone is scarce in the literature, the best-acting sterilant will depend on a myriad of factors and the specific application. Some of these factors include the applied dosage (concentration × exposure time), temperature, humidity, type of microorganisms present, and the material properties of the substrate (porous or nonporous) to be disinfected. A detailed description of these factors is provided by Epelle et al.^45^

**Table 2:**
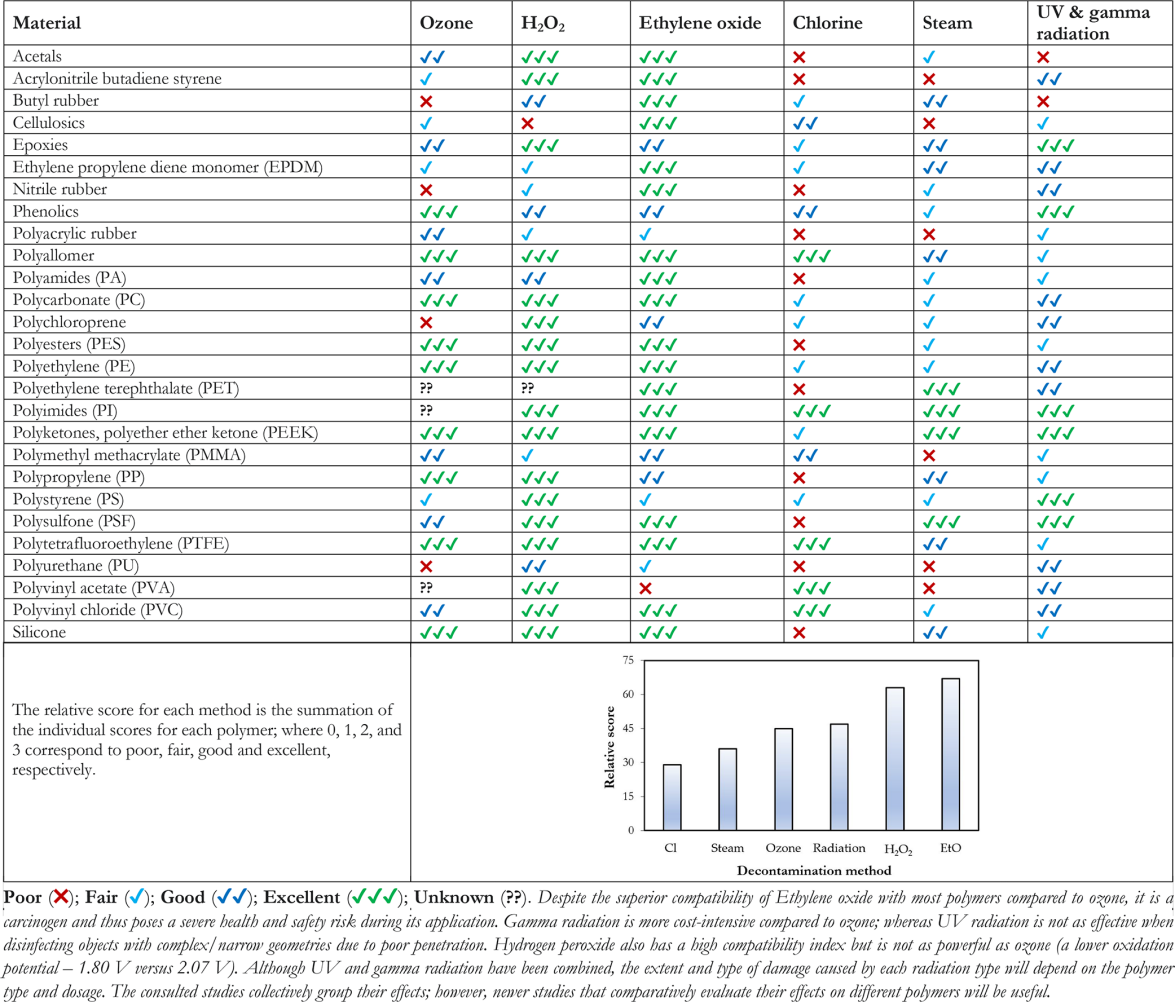
Compatibility of Different Polymers with Commonly Applied Disinfection Methods^25,57,79−84^

As indicated in Table 2, the compatibility of some polymers with ozone is still unknown, an indication of the recency of its application in diverse industries. The most stable materials with excellent resistance to the disinfection methods mentioned in Table 2 include PEEK, PTFE, PVC, and potentially polyimides. Nonetheless, this review table has highlighted that ozone-based disinfection methods are relatively compatible with several popularly applied polymers. Furthermore, hydrogen peroxide also appears to perform better than ozone (Table 2) in terms of material compatibility. Thus, future applications of ozone-based decontamination may involve a hybrid process (O_3_ + H_2_O_2_ + UV + other environmentally friendly gaseous disinfectants).

Depending on the mechanical properties (toughness, ductility, hardness), the desired application, and cost, these material may be further utilized in diverse industries where frequent decontamination is paramount or required. Although mainly polymers are captured in Table 2, metals such as zinc and cast iron can be readily degraded by ozone; stainless steel (SAE 304 and 316) possess better stability against oxidation. The suitability of aluminum tends to depend on the application of the oxidizing agent under wet or dry conditions; wet conditions can potentially cause degradation.

## 3 Engineering Considerations for Deployment of Ozone Technologies

### 3.1 Generation and Decomposition

The splitting of oxygen molecules in air to form ozone can be carried out via ultraviolet (UV) radiation (e.g., 185 nm low-pressure mercury lamps and 172 nm Xenon excimer vacuum UV lamps) or via a high voltage/energy electric field (at low or high frequencies), commonly referred to as corona discharge (CD). These procedures are energy-intensive, and the ozone yield depends on the composition of the feed gas. Utilizing high-purity oxygen (e.g., medical-grade oxygen) as the feed gas can produce 10–15 wt.% ozone concentration, which can be double to quadruple the ozone concentration that can be produced by air.^25,85^ The main disadvantages of mercury lamps are their low UV efficiency, the low absorption coefficient of oxygen, and the simultaneous production of UVC (254 nm), which destroys ozone.^86,87^ Although vacuum-ultraviolet (VUV) lamps principally emit the ozone-producing spectral line, VUV light sources are scarce and tend to have a pulsed operation profile, thus limiting the continuous production of ozone for disinfection.^87^ These limitations, coupled with the higher electrical efficiency of corona discharge methods, have made them more attractive. 1–16 wt.% of ozone can be produced by CD ozone generators compared to 0.001–0.1 wt.% by UV methods; this corresponds to 10–1000-times lower ozone concentration than CD methods.^88^ The specific energy consumption per gram of ozone produced from dry air is 0.515 kWh/g ozone for the UV method (185 nm), whereas it is 0.018 kWh/g ozone for the CD method.^89,90^ Although CD is usually preferred in diverse industrial applications, it has to be fed with clean and dry air or pure oxygen to prevent the formation of nitrogen oxides and corrosive compounds.^91^ During the selection of ozone generators for disinfection applications, it is important to identify if the quoted production rate is based on an oxygen or air feed gas, as this could have massive impacts on the expected performance.^4^

**Figure 2:**
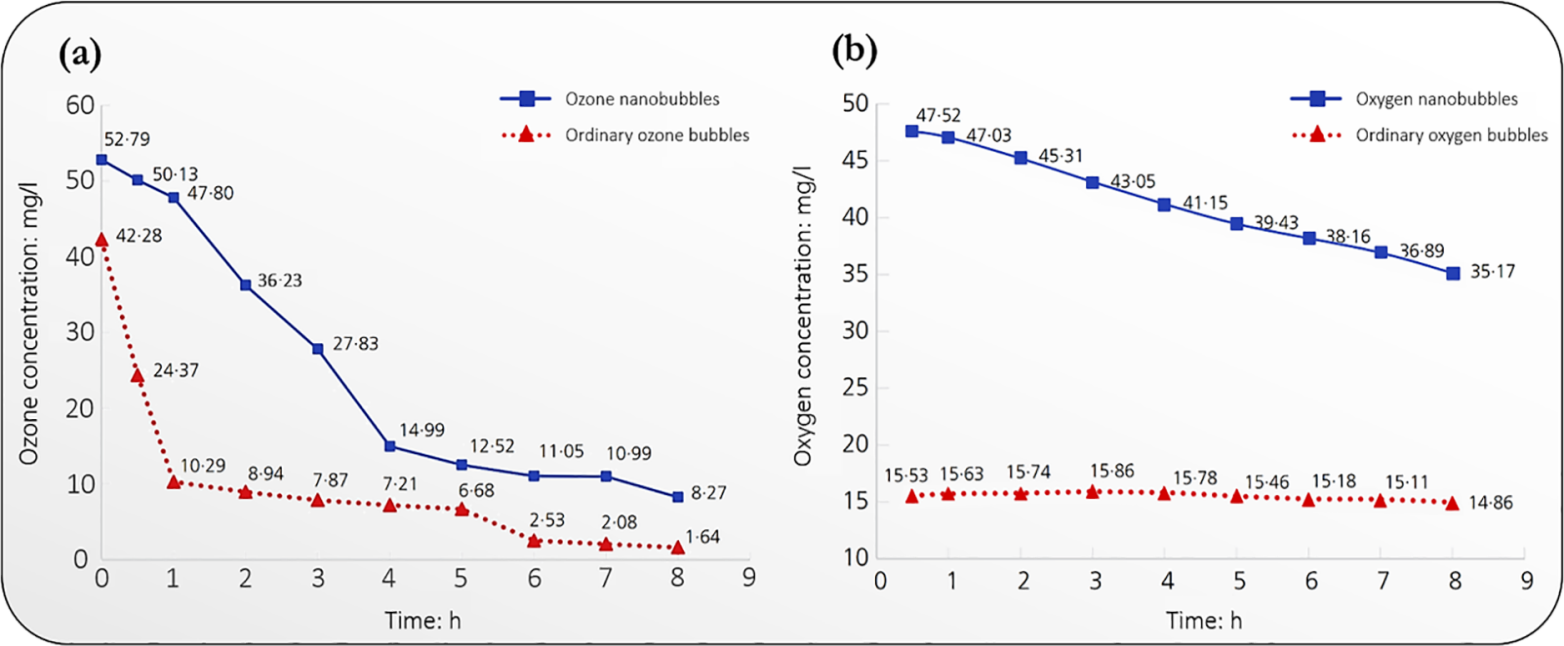
Effect of nanobubble generation over time on (a) aqueous ozone stability and (b) oxygen stability (adapted with permission from ref (92)). Nanobubbles are generally more stable than ordinary bubbles in both scenarios.

Dissolving ozone gas in water requires efficient mass transfer of the gas into the liquid phase, particularly because of the cost involvement of gaseous ozone production (which tends to be higher when high-purity oxygen is used). Venturi injection and bubble diffusion mechanisms have been mainly applied for this purpose.^25^ The former involves the use of a venturi equipped with multiple inlets maintained at a vacuum to facilitate the mixing of the gas and liquid phases via the created pressure difference upon liquid entry into the system. In the latter, pressurized ozone expands via nano/microsized pores on a porous stone into the liquid phase. Acoustic/ultrasound energy, high-intensity light photons in liquids (e.g., UV), and electrolysis are also other applied methods adopted to enhance ozone mass transfer.^92^ It is worth mentioning that nano-, micro- and macro-bubbles may be generated during these procedures; however, it is the smaller-sized nanobubbles that are retained much longer in solution and facilitate ozone decontamination.^93^ Larger-sized bubbles tend to be largely affected by buoyancy, leading to their collapse at the surface, and eventual ozone escape. Figure 2a elucidates the increased stability attainable with ozone nanobubbles compared to ordinary bubbles of larger sizes. Furthermore, the increased dissolved oxygen (Figure 2b) content facilitates the generation of radicals capable of oxidizing pollutants. Rice et al.^94^ highlight some key methods of enhancing ozone mass transfer for laundry applications.

Various studies have reported different half-lives of ozone in air and water. The presence of ozone-consuming compounds in the type of water analyzed is a key determinant of the half-life. While ordinary tap water gave a half-life of 10 min in the study of Epelle et al.,^6^ the half of bottled mineral water was 39 min. Where stable nanobubbles are efficiently produced in ozone-demand-free water, the half-life of ozone may increase even more significantly. Some reports have mentioned stability for weeks of ozone nanobubbles in solution.^95^ The degree of agitation also affects the stability; while this may aid dissolution, continuous stirring must be maintained at an optimal rate, or else increased bubble coalescence may be induced, thus causing a concentration decline via outgassing or ozone decomposition via OH^–^ radical generation during collapse at the surface. Thus, the ozone diffusion mechanism plays a significant role in its stability and ultimate decomposition kinetics. In air, ozone tends to remain longer than in ordinary tap water with conventional bubbling. Half-lives between 20 and 50 min have been typically reported by different authors.^4,96,97^ The application of catalytic ozone decomposition is commonly practised in several industries where spontaneous ozone decomposition is insufficient to meet the peculiar timelines of the process; activated carbon and manganese oxide are two popularly implemented catalysts for this purpose.^98^ Epelle et al. demonstrated that gaseous ozone decomposition over an activated carbon catalyst yields a 24-times faster decomposition rate compared to spontaneous decomposition.^4^ The factors affecting the stability and the corresponding efficiency of microbial inactivation by gaseous or aqueous ozone are numerous. They can be broadly classified into ambient conditions or properties of the disinfection environment, the material or substrate properties, and the operational conditions. The ambient factors involve parameters such as the pH, temperature, pressure, humidity, and dissolved organics concentration; the material or substrate properties include porosity, contact angle, and contamination level; whereas the operational aspects consider the type of organisms, the ozone concentration, exposure duration, ozone generation method, penetrability, and the homogeneity of the ozone distribution in the test chamber/facility. While the impact of the operational factors on the inactivation efficiency can be directly inferred (enhancing the listed factors increases the inactivation efficiency), the influence of the ambient conditions is not as straightforward. Table 3 provides further details on the impact of the ambient conditions (temperature, pressure, relative humidity, pH, conductivity, and the presence of certain additives). The stability of ozone and its inactivation kinetics are determined by these parameters. These parameters can be altered in the disinfection system/environment to facilitate the breakdown of ozone into oxygen. As indicated in Table 3, several studies have also utilized increased temperatures to catalyze ozone decomposition. Decomposition mechanisms of aqueous and gaseous ozone (Table 4) are already well established and can be found in the following studies.^23,98^

**Table 3:** Influence of Key Parameters on Ozone Decontamination and Stability Characteristics^a^

parameter	influence on ozone stability	influence on microbial inactivation
Gaseous Ozone Application		
Temperature	Increased temperature enhances gaseous ozone instability/depletion; at lower temperatures, ozone is more stable.^11,98−101^	Increased temperature increases gaseous ozone’s reactivity, and inactivation efficiency.^11,98,100^
Relative humidity	Increased RH enhances gaseous ozone decomposition.^11,102,103^	Increased RH improves the inactivation efficiency of gaseous ozone.^11,102,103^
Pressure	At pressures lower than atmospheric pressure, the effective lifetime of ozone increases with pressure; at pressures above atmospheric pressure, the effective lifetime of ozone gas decreases with pressure.^101^	Increased pressure (above atmospheric pressure) enhances gaseous ozone inactivation efficiency.^104^ Also, the creation of a vacuum before ozone entry enhances ozone penetration into a porous substrate, thus accelerating antimicrobial action.^105^
Aqueous Ozone Application		
Temperature	Increased temperature enhances aqueous ozone instability/depletion; at lower temperatures, ozone is more stable.^98,100,106^	Increased temperature increases aqueous ozone’s reactivity, and inactivation efficiency.^98,100,106^
Pressure	Increased pressure favors aqueous ozone solubility and stability.^104^	Better stability implies prolonged antimicrobial activity in water.^93^
pH	Aqueous ozone is less stable at high pH.^11,94,107^	Aqueous ozone’s efficacy increases at high pH due to the production of OH^–^ radicals.^11,94,107^
Conductivity	Increasing the conductivity has a dual effect depending on the additive (e.g., structure-breaking or forming salts); there tends to be a threshold beyond which the positive or negative effects may be reversed.^6^	Increasing the conductivity may enhance the inactivation efficiency of aqueous ozone (however there tends to be a threshold beyond which the effect is reversed).^6^
Bubble size	Nanobubbles stay in solution longer and promote ozone stability compared to macrobubbles, whose collapse aid ozone decomposition.^92,93,108^	Reduced bubble size enhances mass transfer and induces better inactivation by aqueous ozone^92,93,108^
Viscosity	Increasing the viscosity of the solution enhances ozone stability, delaying its decomposition.^109,110^	The microbial inactivation efficiency of the solution is retained for a long time at high viscosities.^109,110^
Additives (surfactants)	Controlled surfactant concentration may favor ozone stability.^6,107^ However, this may significantly consume ozone in solution beyond a certain threshold, which could be the critical micelle concentration for some surfactants.	Inactivation is enhanced by the joint action of the surfactant.^6^

a It is important to note the decomposition of ozone, particularly in aqueous and humidified environments leads to the formation of hydroxyl radicals, which have a higher oxidation potential than ozone itself. These radicals are capable of providing additional antimicrobial properties (indirect oxidation). Hence, the rate of formation and disappearance of hydroxyl radicals, which also depend on these listed factors, ought to be considered together with the direct oxidative effects of ozone, for any system.

**Table 4:** Comparison of Practical Considerations Required for Gaseous and Aqueous Ozonation^a^

factor	ozonation in air	ozonation in water
Need for drying	Substrate is usually dry, eliminating the need for further drying after treatment.	Drying after treatment is required since the substrate becomes wet, particularly if porous (e.g., textiles).
Cleaning	Does not clean the substrate; only disinfects or sterilizes it. A separate cleaning step is required and is best to carry out before ozonation.	Cleaning and disinfection may occur simultaneously. Furthermore, the use of surfactants can promote aqueous ozone stability.^6,107^
Limitations to ozone generation	Higher ozone concentrations (e.g., up to 50 ppm) can be attained rapidly; this depends on the capacity of the generator and volume of the chamber.	Attainable ozone concentration is limited by mass transfer factors, relative to gaseous ozonation, for the same volume and generator capacity.
Concentration homogenization	Efficient gas circulation systems are required for concentration homogenization.	Concentration homogenization strongly depends on efficient gas dispersion in water; this may cause high gas usage.
Penetration efficiency	Hard-to-reach areas of the substrate can be better disinfected due to increased penetration of gaseous ozone.	Liquid penetration efficiency may be adversely affected for certain substrates with difficult geometries (e.g., small-diameter endoscopes).
Parameters influencing ozone stability	Humidity and temperature are the key influencing factors on the stability of ozone.	The stability of ozone during a treatment cycle is a function of many variables (pH, conductivity, temperature, pressure, water composition and ozone demand constraints).
Safety	Ozone’s detrimental impact on human health, implies airtight ozone chambers are required if the ozone equipment is to be located in an inhabited area.	Ozone’s impact on human health is significantly reduced when it is dissolved in water.^35^

a Adapted from Epelle et al.^7^

### 3.2 Disinfection Byproducts

The unintended formation of persistent transformation products and disinfection byproducts (DBPs) particularly during aqueous ozone application is a key environmental challenge, especially during immersive/aqueous treatment of medical devices or during wastewater treatment. They can be formed as a result of the reaction of ozone with dissolved organics and some inorganic compounds present in the utilized water and can be difficult to eliminate.^111^ The type of DBP produced and its toxicity depend on the original composition of the water. For example, bromide-containing waters will produce bromate during ozonation, and this is a human carcinogen. Additionally, the myriad of DBPs that could be formed, with unknown specific toxicities and health implications,^22,112^ is a prevalent source of concern. Although the most common toxicological behavior of DBPs is carcinogenicity, some of them could be neurotoxic, mutagenic, cytotoxic, teratogenic, and possibly genotoxic, with several adverse outcomes on human health.^113^ While harmful DBPs are mostly described as a challenge of aqueous ozone application, the radicals generated during gaseous ozone generation and decomposition may also induce the formation of partially oxidized DBPs particularly under humid environments and in the presence of UV radiation and volatile organic compounds (VOCs);^114^ however, these tend to be short-lived. The production of nitrogen oxides (NO_x_), another byproduct, during the generation of ozone from air (instead of pure oxygen)^115^ can be problematic at high concentrations. Exposure to high levels of NO_x_ can damage the respiratory airways; the Occupational Safety and Health Administration (OSHA) has set a permissible exposure limit (PEL) of 25 ppm for nitric oxide and a short-term exposure limit of 1 ppm for nitrogen dioxide. Gas-phase nitrous acid (HONO), which may be formed under these conditions, has been identified as an emerging pollutant.^116^ This species is known to be a major photolytic source of hydroxyl radicals in air.^117^

The application of hybrid methods (advanced oxidation processes) is a popularly applied route toward mitigating the formation of DBPs.^118^ Thus, proper characterization of water composition and rigorous kinetic studies are required (as part of the design phase of treatment facilities) to predict the potential formation of DBPs or harmful transformation products, their concentrations, and removal methods before and after the application of ozone for treatment.^119^ Additionally, appropriate control of the ozone dosage (based on the microbiological and environmental requirements) should be carried out to avoid unnecessarily high production rates of ozone gas. Multiple passes through effective decomposition catalysts and constant concentration monitoring of potentially harmful DBPs in the ozone treatment environment should be carried out.

### 3.3 Scalability and Flexibility

Before large-scale implementations of ozone disinfection systems are started, lab-scale experimentation on the required dosage is inevitable. Upscaling lab-scale conclusions to large systems should be done with care, particularly when differences in material type for chamber construction and ozone demand in the immediate environment are expected. However, the study by Zoutman et al.^120^ demonstrated the scalability of ozone disinfection systems (obtaining similar inactivation results in a small test chamber, 0.25 m^3^, and in a large room, 82 m^3^). Furthermore, for certain applications where there is the inherent flexibility of choosing the ozone disinfection medium (gaseous or aqueous), Table 4 provides a list of factors to consider before this choice and the corresponding investments are made.

It has been recently reported that gaseous ozonation (*T* = 18 °C and RH = 50%) can be more effective than aqueous ozonation (*T* = 18 °C), particularly when disinfecting wet porous substrates at the same ozone concentration and exposure duration.^7^ In their study, the decontamination of *S. aureus* gave the reverse observation of all organisms tested. A similar observation was also made by Martinelli et al.^121^ However, Megahed et al.^122^ reported the superiority of aqueous ozone treatment over gaseous treatment of nonporous substrates contaminated with cattle manure. Similar observations are also reported by Tizaoui et al. against the SARS-CoV-2 virus.^103^ In these studies, the microbes were mainly dried onto the surfaces of the nonabsorbent materials utilized. This indicates that the nature of the substrate (porous or nonporous and wet or dry) and the type of microorganisms present affect the performance of gaseous and aqueous ozonation. The respective contributions of direct and indirect oxidation during gaseous (humid or dry) and aqueous ozonation are also attributable to these observations. Thus, it is important to establish the required dosage threshold for the specific application involved before applying each ozonation method.

### 3.4 Automation of Ozone Systems

The advent of the COVID-19 pandemic led to several developments of automated gaseous ozone disinfection systems for a variety of materials. Typical ozone disinfection cycles comprise the ozone generation duration, the stabilization duration or dwell time for ozone to act at the desired concentration, and the decomposition during which ozone is converted to oxygen spontaneously or via a catalyst.^5^ The goal of automating this process is to reduce the total cycle time attributable to the load, disinfect, and unload process. Furthermore, automation reduces the need for manual handling, which could cause further contamination post treatment. Recently, Rodriguez^123^ developed an automatic disinfection device that comprises an object conveyor that transports items to and from a cabin that houses an ozone generator. They proposed the use of sash doors to completely isolate the internal section of the cabin to prevent ozone exposure. The cabin consists of 3 compartments, where the 2 end chambers are fitted with an ozone decomposition catalyst. Another invention by Silla et al.^124^ for the disinfection of apparel using ozone gas also featured a conveyor, with a system of automatic doors and an optional heating system to aid ozone decomposition after the main treatment cycle. The invention by Miller,^105^ particularly for mail articles involves a similar 3-chamber plus conveyor arrangement (with an optional single chamber scenario) and is equipped with a vacuum pump to enable efficient penetration of ozone gas into the contaminated articles.

A recently developed automated ozone system (patent filed) by ACS Clothing Ltd. for garment decontamination utilizes the slower decomposition rate of ozone in air (relative to aqueous ozone) for continuous decontamination of clothing items and PPE. The items are fed by a conveyor into a chamber with a preset ozone concentration. This concentration is maintained by a control system that regulates the ozone generators’ outputs depending on the concentration set point (as determined by extensive experimentation). Thus, the cycle time is cut down significantly by eliminating the need for continuous ozone decomposition and regeneration from scratch. An interconnected system of conveyors, multiple chambers, and air curtains is applied to prevent the escape of ozone from the system. By applying an ozone dose consisting of a high exposure concentration and shorter exposure duration, thousands of garments can be treated within an hour. From the experience of the authors, a logistics problem that has arisen from embedding automation is matching the high throughput of the disinfecting chamber with other workstations during garment processing. To resolve this, the cycle time in the disinfection chamber was increased, and the ozone concentration was lowered while maintaining the required dosage. This way, the potential for a bottleneck at the next station (usually the bagging machine) is effectively managed. As suggested by Farooq and Tizaoui,^8^ lower ozone concentrations for longer durations, are likely to give the same inactivation efficiency as high concentrations over shorter durations (so far the same dose is administered). This excellent flexibility provided by ozone, makes it very attractive, particularly for ensuring worker safety and solving logistical problems during industrial implementation.

There have also been several developments in the application of ozonated water sprays for the disinfection of different articles, employing varying degrees of automation. One of these developments is that by Maurya et al.,^125^ where an autonomous disinfection spray tunnel was applied to disinfect external surfaces at the peak of the pandemic in India. The interested reader is referred to the work of Mascarenhas et al.^3^ which provides a detailed overview of patented inventions on ozonated water spray devices. However, conveyor systems, automatic doors, and automatic ozone level control systems are all common features of these inventions. These advances and several others^35,126^ will better position relevant authorities, and organisations to prevent or effectively combat future pandemics.

For aqueous ozone application, it is also important to highlight the necessity of robust control systems for the ozone concentration, as this avoids ozone overdose/oversaturation, which is could cause loss of ozone gas to the environment above the air–water interface. Although the oxidation–reduction potential (ORP) has been frequently utilized^127^ as the control parameter for this purpose, it is an indirect measure (which considers multiple oxidants, including ozone), thus making it difficult to ascertain the actual amount of ozone required to treat the immersed object.^128^ However, the use of the ORP may be attributed to the fact that the presence of other oxidizing agents may affect the microbial inactivation efficacy. Nonetheless, direct control of ozone concentration is mostly desirable.

### 3.5 Circulation and Extraction

**Figure 3:**
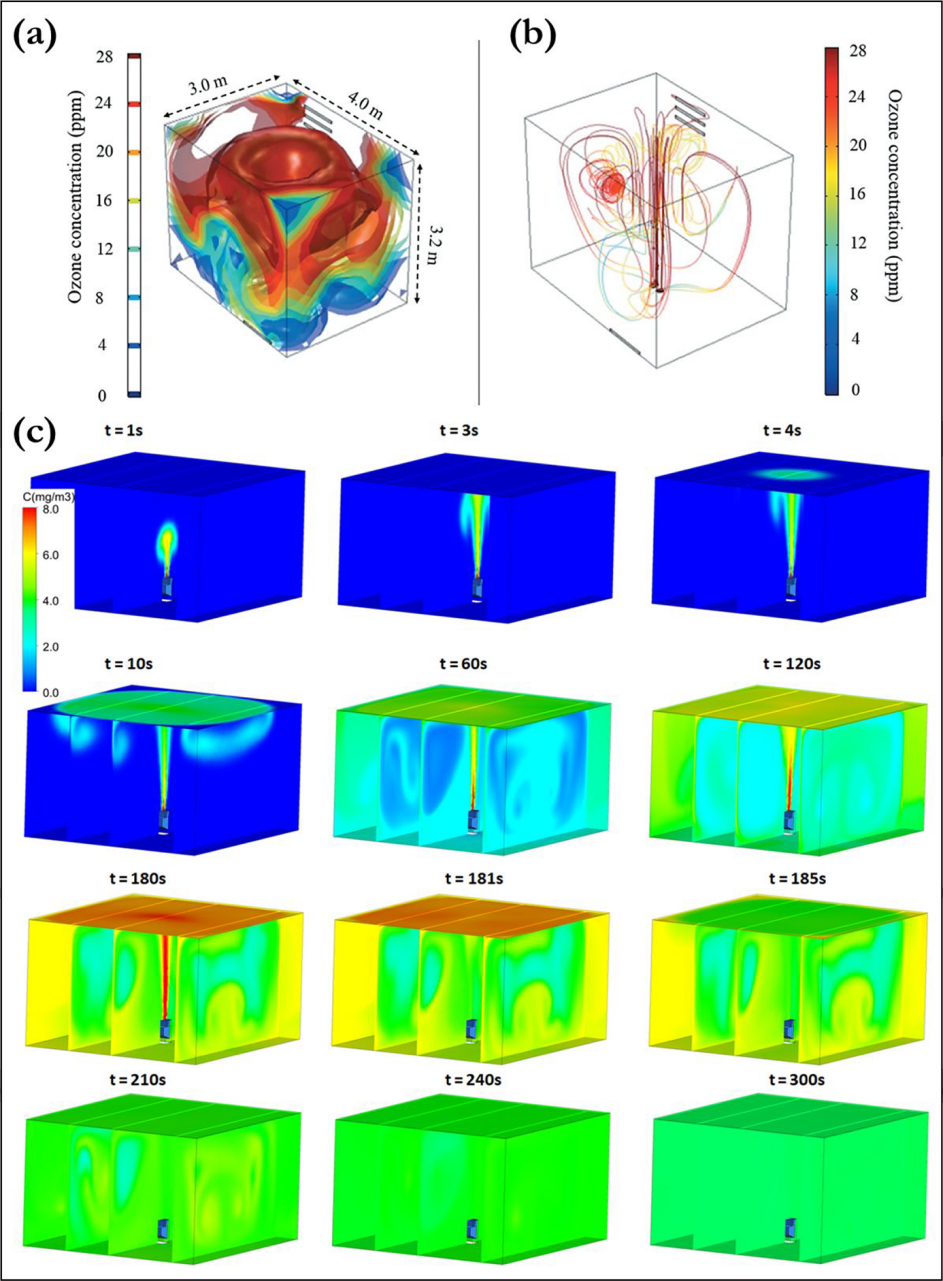
Application of computational fluid dynamics (CFD) to analyze the circulation of ozone gas in a room for the decontamination of surfaces and objects. (a, b) Contours and streamlines of ozone concentration using COMSOL Multiphysics in the work of de Souza et al.;^129^ (c) time variation of ozone spread in a room via contour plots on different planes using ANSYS Fluent.^130^

Given the high relative density of ozone gas (1.7), adequate circulation systems are required to ensure proper contact with the objects and surfaces of interest to be disinfected. This is particularly important for objects of complex geometries and for large-scale applications, where several items are to be disinfected. Computational fluid dynamics (CFD) becomes an important tool to apply for this purpose. As demonstrated in Figure 3a and b, de Souza et al. applied finite element CFD simulations using COMSOL Multiphysics to examine ozone’s spread in an office via an ozone generator.^129^ The validated model enabled the authors to identify regions of the room with low concentrations, due to poor mixing. A similar study by Jarohmi et al.,^130^ shown in Figure 3c, utilizes the scalar transport model in Ansys Fluent (a finite volume solver) to model the dispersion of ozone gas from a generator in a room. Excellent agreements with experiments were observed in their work. The conditions shown in Figure 3c represent a generation time of 3 min (at 7 g/h) followed by a 2 min dwell time. CFD studies of this kind provide insights into the number of generators required based on the desired concentration level desired, and the optimal locations of the generators and circulation systems to ensure adequate ozone interaction with the items to be decontaminated. CFD methods can also be applied to study ozone fogging or ozone misting operations, using Eulerian-Eulerian or Lagrangian-Eulerian multiphase flow models.

**Figure 4:**
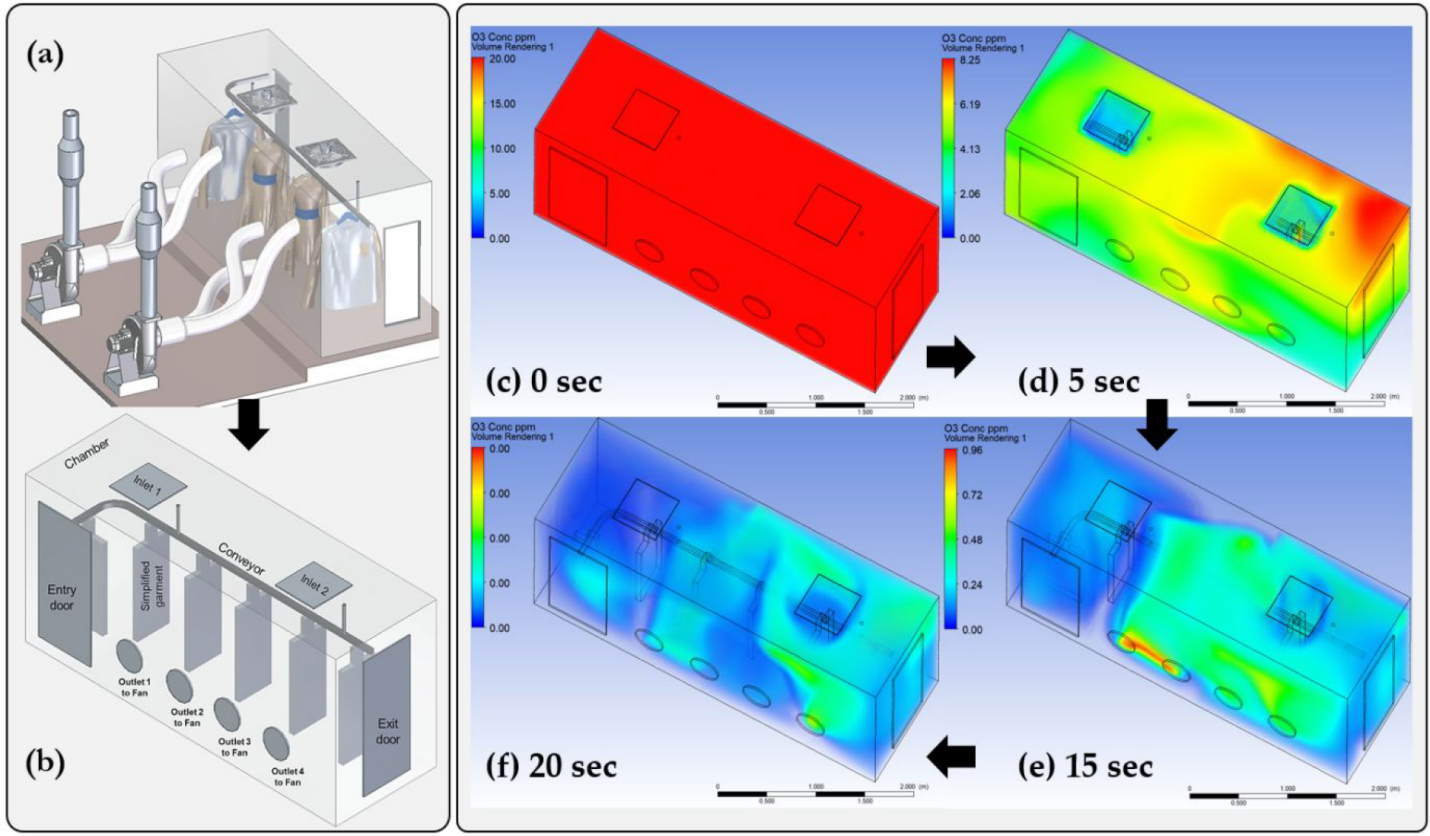
Application of computational fluid dynamics (CFD) to analyze the removal rate of ozone gas from a clothing disinfection chamber using extraction fans; (a) chamber configuration, (b) simplified 3D model (to reduce the number of meshing elements) used for the CFD simulation in ANSYS Fluent; (c–f) time variation of ozone removal from the system.

During emergency procedures, it may be required to rapidly extract ozone gas from the chamber (through a catalyst) to ensure worker safety. As an example, Figure 4 shows a downscaled simulation of an ozone chamber, which the authors have designed for industrial disinfection applications. The pressure drop through the catalytic destruct units at the fan outlets (Figure 4a), the fan static pressure drop, the attainable air flow rate, duct size, and the pressure at sealable inlets are key factors affecting the efficiency of ozone removal rates from the system. Numerical simulations of an ozone misting process for the sanitization of hospital facilities have been performed by Schroer et al.^131^ Their validated CFD model enabled an accurate distribution of the ozone mist concentration to be obtained. Besides the application of numerical CFD computations, other important analytical models to bear in mind when designing ozone disinfection systems are shown in Table 6.

### 3.6 Measurement of Ozone Concentration

The measurement methods of ozone in gas and water are already well-established in the literature.^25,29,91^ Fourier transform infrared spectroscopy (FTIR) is often used for gaseous ozone measurement; this method is however not suitable for aqueous ozone measurements.^72^ UV absorption has also been extensively used and is adaptable to both gaseous and aqueous ozone measurements. Nonetheless, organic compounds in culture media may limit their applicability.^72,132^ Pang et al. reported that most UV-based methods for gas-phase measurements require high amounts of power and sampling gas flow rates; they also tend to be expensive.^133^ Sensors based on gas-sensitive semiconducting oxide technologies are also commonly used.^134^ Electrochemical ozone sensors can produce a voltage signature between an anode and cathode that correspond to the amount of ozone present.^25^ They usually involve the use of a porous membrane that allows ozone to pass through into a cell containing electrodes and an electrolyte. The contact of ozone and the electrolyte causes a change in the electrochemical potential between the electrodes causing a flow of electrons. This method is robust and can be used for both gas and liquid phase measurements but may be expensive and require frequent calibration.^6^

Aqueous ozone can be readily measured via colorimetric methods such as the *N*,*N*-diethyl-p-phenylenediamine (Palin DPD) method and the indigo method. The oxidation of the iodine ion in a DPD + KI buffered solution causes a pink coloration whose relative absorbance can be measured spectrophotometrically.^29^ Similarly, ozone interacts with the carbon-double bonds of sulfonated indigo dye to decolourise it, and the change in absorbance gives an indication of the ozone concentration.^91,135^ However, these methods tend to be affected by other oxidizing agents present in the solution (e.g., Cl^–^, Br^–^ Mn^2+^, OH^–^).^29,72^ Palin DPD method implements a correction for the presence of Cl^–^, Br^–^ and in solution using glycine tablets.^136^ However, this increases the difficulty of continuous data collection, which is often required for decomposition kinetics studies. Although these colorimetric methods tend to be cheaper than electrochemical methods, they are not as robust. More recently, Wright et al.^72^ highlighted Pittsburgh Green fluorescence probes as being sensitive and specific to ozone. Although not commercially available, it has considerable potential to be widely applied if further developed. The interested reader is referred to the work of Korlu et al., which provides a good overview of aqueous measurement techniques not covered herein.^29^ In summary, electrochemical methods, although costly, provide accurate and robust ozone concentration measurements. Whichever ozone-sensing method is adopted, extensive and reasonably frequent calibration should be performed.

### 3.7 Material Selection, Health, and Safety

Given ozone’s degradative properties, construction materials must be carefully selected during the design and installation of gaseous or aqueous ozone equipment. Cost-effective metals like aluminum may be affected by moist conditions during ozone treatment.^137,138^ It is also important to pay close attention to the grading of steel materials to be used (stainless steel 304 and 316 are the most resistant to ozone degradation). As shown in Table 2, several popularly applied polymers have limited compatibility with ozone; thus, critical consideration is required for the material selection phase of any design endeavor involving ozone. Routine checks are also important to ensure that installed safety systems (e.g., rubber seals) are not compromised due to the degradation of the materials as a result of ozone application. While we concentrate on the resistance of several polymers (which are often degraded via chain scission and the breakage of cross-links) in Table 2, the ozone resistance of commonly utilized metals can be found in ref (138).

The extent of ozone degradation of highly unsaturated polymers tends to be higher than that of saturated polymers, according to a study by Giurginca et al.^139^ Using IR spectra and kinetic data, they established that ethylene-propylene-diene elastomer is more susceptible to ozone attack as a result of the presence of double bonds in the macromolecules. A study^140^ on the degradation of a high-temperature epoxy showed an ozone oxidation depth of up to 120 μm. Exposure of the neat resin to 1% of ozone for 3 months at room temperature showed that cross-linking dominated in the first week, resulting in a slight stiffening of the polymer. However, as aging continued, a chain scission mechanism became dominant, resulting in a reduction of the load to failure. This demonstrates the intricate relationship between ozone degradation and the chemistry of the polymer, and thus the increased necessity for regular inspections since ozone’s impact on certain polymers is not always a progressive deterioration.

With ozone disinfection systems (particularly gaseous ozonation), there is a need for continuous monitoring. It is quite often the case that the detection limit increases with the maximum measurable concentration of the sensor. Thus, large-scale ozone applications that utilize high concentrations of ozone require sensors capable of detecting ozone concentrations within the occupational exposure limit values. In the UK, the short-term (usually 15 min) worker exposure level for ozone is 0.1 ppm (Table 5). Grignani et al.^141^ present a comprehensive list of the exposure levels in different countries. Ozone causes severe irritation of the respiratory tract as well as lung damage; coughing and chest tightness are characteristics of uncontrolled exposure to ozone.^100^ There has been some discussion throughout the COVID-19 pandemic regarding the use of low ozone concentrations in occupied spaces to reduce the risk of disease transmission. However, the intentional generation of ozone in occupied spaces for this purpose is not encouraged. Table 5 presents the exposure limits of ozone relative to other gaseous disinfectants.

**Table 5:** Exposure Limits of Some Gaseous Disinfectants^a^

disinfectant	exposure limits	ref
Ozone	0.1 ppm (OSHA-PEL-TWA)	(142)
	0.3 ppm (Cal/OSHA-PEL-STEL)	
	0.1 ppm (NIOSH-REL-C)	
Glutaraldehyde (GTA)	0.05 ppm (ACGIH-TLV-TWA)	(143)
	0.2 ppm (NIOSH-REL-C)	
Peracetic acid	0.4 ppm (ACGIH-TLV-STEL)	(144)
Hydrogen peroxide	1 ppm (OSHA-PEL-TWA)	(145)
Ethylene oxide	1 ppm (Cal/OSHA-PEL-STEL)	(146)
Chlorine	1 ppm (OSHA-PEL-TWA)	(146, 147)
	1 ppm (OSHA-PEL-C)	
	0.5 ppm (NIOSH-REL-C)	
Ortho-phthalaldehyde (OPA)	–	–

a PEL: Permissible exposure limit; STEL: Short-term exposure limit (usually 15 min); TWA: Time-weighted average over an 8-h shift; TLV: Threshold limit value; REL: Recommended exposure limit; C: Ceiling; OSHA: Occupational Safety and Health Administration; ACGIH: American Conference of Governmental Industrial Hygienists; NIOSH: National Institute for Occupational Safety and Health; OPA is generally considered safer that GTA; however, exposure limit data for this disinfectant is scarce; **—**: No data.

### 3.8 Economics of Large-Scale Disinfection Systems

The cost of a large-scale ozone disinfection system is typically dependent on the intended application (water treatment, textiles disinfection, medical equipment sterilization, etc.) and the capacity of the disinfection facility. Furthermore, the desired ozone dose affects the runtime and capacity of the ozone generators, which in turn affects electricity consumption and operating costs; as previously highlighted, ozone generation is an energy-intensive process. Data provided by Champion Technology in 1998 suggest a capital expenditure, (CAPEX) > $250,000, for the treatment of 1 million gallons of wastewater per day (which had undergone pretreatment), and an annual operating expenditure (OPEX) > $18,000.^148^ The presented analysis concluded that the costs are site-specific and depend on the plant’s effluent limitations. Rice et al.^149^ provided a comparative assessment of capital and operating ozonation costs for drinking water treatment plants in Belgium, Switzerland, France, and the US. For a 687 ML/day plant in Belgium, a capital cost of $4,024,000 was stated, whereas the electrical cost was ¢3.01/kWh. Similar electrical costs were quoted for different regions. Remondino and Valdenassi^18^ presented a case study of ozone’s therapeutic application in animal husbandry (specifically, a pig farm). They reported a €90,000 cost for purchasing the ozone plant, whereas, between €5,000 and 6,500 is incurred per year for the plant’s maintenance (€4,000 of which are related to electrical costs). ACS Clothing Ltd., a clothing rental and fulfilment company in Scotland UK, is on the verge of completing the installation of an automated (semicontinuous) gaseous ozone disinfection system for garments. The system, which is capable of disinfecting 20,000 garments within an 8-h shift, involves an approximate investment of £270,000. Ozone generators, ozone sensors, pin and clip conveyors, disinfecting and housing chambers, air curtains, extraction fans, circulation fans, ducting, catalytic destruct beds, and the control system/software are the main cost components, with the automatic conveyor systems constituting approximately 32% of the CAPEX. A thorough economic analysis of this system is presented in a separate study by the authors.^45^

### 3.9 Mathematical Models

Table 6 highlights some key equations and models to consider when determining the optimal generation capacity for a particular system. One key question that transcends several industrial applications involving the utilization of gaseous ozone generation is the length of time to operate an ozone generator of a certain capacity to achieve a desired concentration level. Equation 4 (Table 6) provides a guide toward developing a reasonable estimate of the operational time. Since ozone autodecomposes to oxygen, the knowledge of the ozone generator performance coupled with the half-life (eqs 7 and 8) of ozone in the specific environment of interest can facilitate the design of an optimal control strategy that maintains the ambient concentration at a set value. Furthermore, the table includes the inactivation kinetic correlations of different organisms, which allows a design team to make recommendations on the length of the treatment cycle for effective inactivation. The electrical energy requirements as well as the diffusion rate and attainable concentration during aqueous ozone application are also presented. It is important to mention that **t**he models in Table 6 do not fully consider important design concepts such as complex gas–liquid mass transfer effects and ozone gas dispersion and penetration, the impacts of which may be adequately studied using computational fluid dynamics (CFD), as presented in Figures 3 and 4.

**Table 6:**
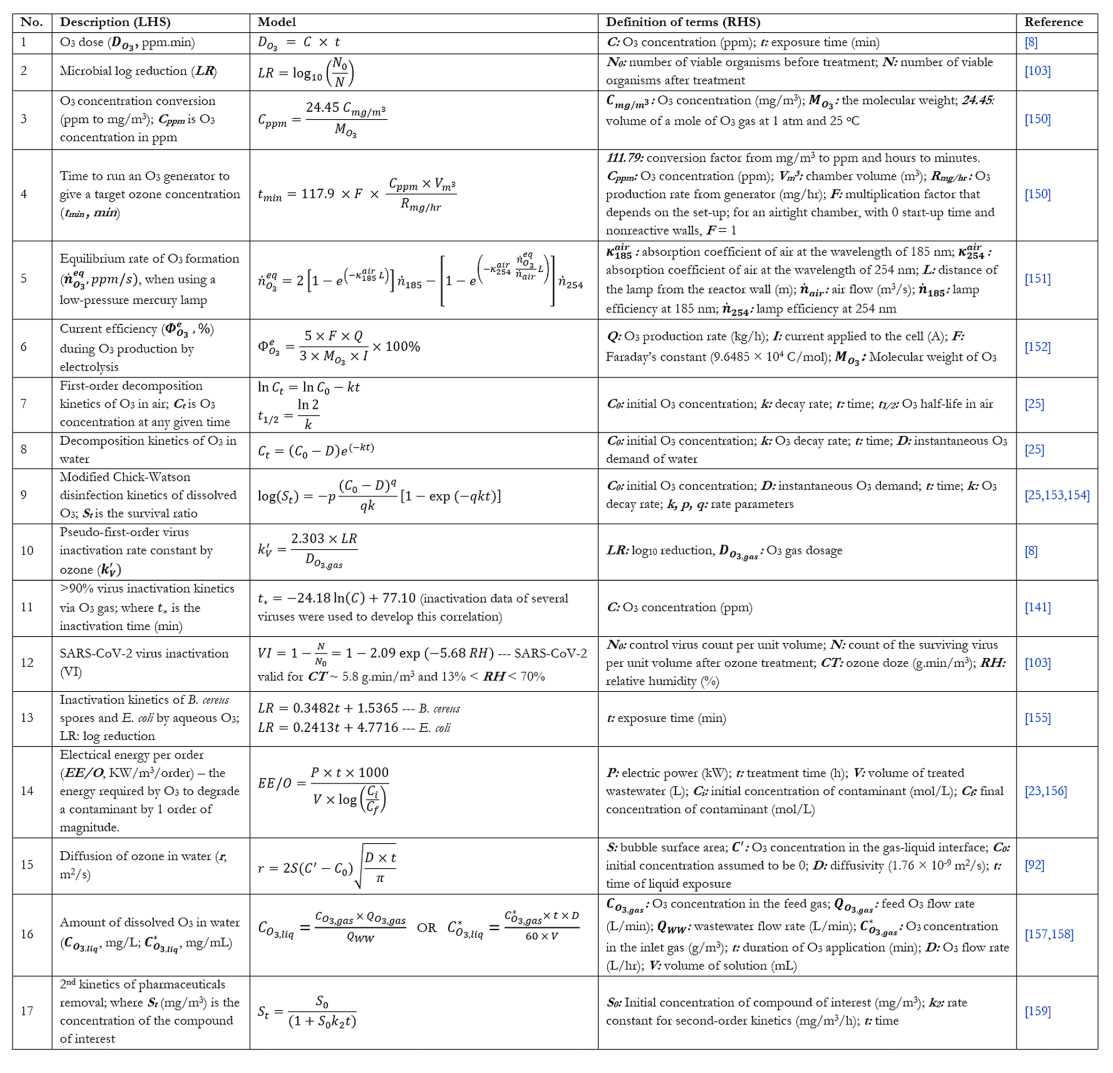
Relevant Models and Correlations to Consider When Designing Aqueous and Gaseous Ozone Decontamination Systems−

## 4 Open Research Problems, Opportunities, and Recommendations

In this section, we highlight some of the key gaps in the literature that warrant further development. Additionally, we also draw inferences from our industrial experience of implementing industrial-scale aqueous and gaseous ozone disinfection systems in providing the following recommendations for future exploration.

Life-cycle assessments (LCA) and environmental impact assessments are required for large-scale ozone systems to quantify their benefits relative to other popularly applied decontamination methods. More research efforts are also required to demonstrate the scalability of different disinfection methods; this can be achieved by comparing the disinfection efficiency attainable within the lab-scale test chamber with that in a large room under the same dosage conditions.

Automated and rapid ozone disinfection systems require further development for throughput and safety enhancement in industrial operations. New advancements in the design of robust control systems for the accurate regulation of ozone concentration levels in air and water are required. Some studies rely on the ORP (mV), which is not a direct implication of the disinfection ability of ozone. Comparative studies that examine the additional compounds generated by industrial and domestic ozone generators will help enhance personnel safety. Furthermore, a systematic quantification of the ozone generation efficiencies when using medicinal oxygen and ambient air should be elucidated. A direct comparison of the efficacies of EtO, hydrogen peroxide and ozone (at the same set of conditions) is also required to evaluate the inactivation kinetics and performance characteristics of these methods.

As highlighted in Table 2, the compatibility of some materials (e.g., PET and PVA) with ozone is yet to be determined. This demonstrates the relatively recent application of ozone for the sterilization of devices made from these materials. A detailed ozone-compatibility assessment of these materials is necessary. The ozone-adsorbent capability of some polymers (e.g., polystyrene^160^), and their subsequent efficient release, enhances the biocidal properties of surfaces made from this material. Further exploration into other polymers or classes of materials (e.g., zeolites) with a similar potential is required as they hold great potential for the development of self-disinfecting surfaces. However, this seemingly advantageous attribute, may also pose a health risk during ozone disinfection of reusable medical devices (particularly respiratory devices), if ozone is not totally removed after treatment; this adsorbing and subsequent release attribute deserves further investigation for a variety of polymers. In addition, there appears to be some conflicting information regarding the resistance of aluminum to degradation over prolonged ozone exposure. More clarity is required in this regard, considering the prevalence and cost-effectiveness of this metal for constructing large-scale chambers.

As demonstrated by Epelle et al.,^4^ the ozone decomposition rate via activated carbon catalyst is approximately 24-times that of ozone’s natural decomposition. Further improvements in catalytic ozone decomposition will help ensure cycle time reduction and operational safety in automated ozonation systems. More developments are also required on the stabilization of ozone in water via viscosity enhancement of the solution. Although glycerol has been successfully applied, further studies utilizing safe and environmentally friendly polymers are needed. These may be useful as hand sanitizers, a friendlier alternative to ethanol-based sanitizers. Alkyl polyglycosides for example can be investigated in this regard; the OH groups present in their structure may also enhance the biocidal action of ozone. For certain applications as in ozone therapy, the release rate of ozone is key to achieving the desired efficacy. The identification and development of materials capable of controlled ozone release in aqueous and dry environments is essential. Furthremore, hybrid oxidation-based methods (e.g. O_3_ + H_2_O_2_) may be investigated as potential routes to attain the high material compatibility of EtO; thus enabling its replacement for the rapid sterilisation of reusable medical devices.

Further analyses of ozone decontamination of textile materials are required to demonstrate its effectiveness in accordance with established standards such as the European Standard BS EN16616.^161^ The benchmarking of ozone’s performance against these standards (applying the recommended microorganisms, and required contamination levels) will further facilitate its large-scale adoption. The textile industry will also benefit from the application of ozone in the mist form (dry fogging), particularly where the textile materials are required to be dry, post-treatment, and where material compatibility or additional cost concerns limit the thorough application of gaseous ozone.

## 5 Conclusions

This review summarizes key engineering factors to consider in the design and implementation of ozone decontamination systems. While several successful lab-scale demonstrations of ozone’s effectiveness against a myriad of microorganisms exist in the literature, details of large-scale deployments of ozone technology are lacking. Factors such as the medium of application (air or water), material compatibility, efficient circulation and extraction, measurement and control, automation, scalability, and process economics must be carefully considered in the design and implementation phases of industrial ozone decontamination systems. Nonetheless, we present some progress made by the authors on the application of automation technologies to ozone systems for the disinfection of clothing and PPE items. The compatibility of ozone with several polymeric materials as shown in Table 2 also appears to be a key determinant of its applicability, despite its widely acknowledged antimicrobial efficiency. The evaluation of concentration thresholds over a repeated number of cycles for a variety of popularly applied materials with limited ozone compatibility will go a long way toward mitigating potential degradation. The application of hybrid ozonation methods, particularly green methods, with no toxic residues (e.g., UV + O_3_, H_2_O_2_ + O_3_, Peracetic-acid + O_3_) also holds great potential for addressing these compatibility constraints, without compromising the disinfection efficacy and material compatibility. It is hoped that the insights presented in this study will support the transition from carcinogenic ethylene oxide to ozone- and hydrogen peroxide-based methods, for the sterilization of medical devices and other materials—a current and global innovation challenge.

## Abbreviations

ACGIH: American Conference of Governmental Industrial Hygienists
CAPEX: Capital expenditure
CD: Corona discharge
CFD: Computational fluid dynamics
CFU: Colony-forming unit
EtO: Ethylene oxide
LCA: Life cycle assessment
NIOSH: National Institute for Occupational Safety and Health
OPEX: Operating Expenditure
OSHA: Occupational Safety and Health Administration
PET: Polyethylene terephthalate
PPE: Personal protective equipment
STEL: Short-term exposure limit
TLV: Threshold limit value
TWA: Time-weighted average
